# Phylogeography and karyotypic evolution of some *Deuterodon* species from southeastern Brazil (Characiformes, Characidae, Stethaprioninae)

**DOI:** 10.1590/1678-4685-GMB-2023-0044

**Published:** 2023-07-10

**Authors:** Igor Henrique Rodrigues-Oliveira, Pierre Rafael Penteado, Rubens Pasa, Fabiano Bezerra Menegídio, Karine Frehner Kavalco

**Affiliations:** 1 Universidade Federal de Viçosa, Laboratório de Genética Ecológica e Evolutiva, Rio Paranaíba, MG, Brazil.; 2 Universidade Federal de Minas Gerais, Instituto de Ciências Biológicas, Belo Horizonte, MG, Brazil.; 3 Universidade Federal de Viçosa, Laboratório de Bioinformática e Genômica, Rio Paranaíba, MG, Brazil.; 4 Universidade de Mogi das Cruzes, Centro de Pesquisas Tecnológicas, Mogi das Cruzes, SP, Brazil.; 5 Universidade de Mogi das Cruzes, Centro Integrado de Biotecnologia, Mogi das Cruzes, SP, Brazil.

**Keywords:** 5S rDNA, 18S rDNA, chromosomal polymorphisms, molecular clock, phylogeny

## Abstract

*Deuterodon* is a genus of the subfamily Stethaprioninae, a group of Neotropical fish known as tetras. *Deuterodon hastatus* represents a species complex, which is supported by cytogenetic and molecular data. In this study, we show the results of comparative evolutionary analyses of the *ATP synthase subunit 6* gene in four *Deuterodon* species, in addition to ribosomal markers (*18S rDNA* and *5S rDNA*), of a new population of the *D. hastatus* species complex from the Angra dos Reis/RJ region. The study population comprised a new cytotype, which we refer to as cytotype D, in *D. hastatus*, with 2n = 50 = 6M+8SM+8ST+28A. We obtained three different clades of *D. hastatus* in our phylogeny, one of them composed only by specimens of cytotype D. By using molecular clock dating, we observed that the radiation of *Deuterodon* from southeastern Brazil seemed to be associated with neotectonic events that occurred during the Miocene-Pliocene and Pliocene-Pleistocene transitions, marked by the capture of headwater streams and marine transgressions. The results obtained reinforce the idea that *D. hastatus* is a species complex, and at least three evolutionary significant units were identified in this group.

## Introduction

*Deuterodon* is a genus of small fish, known as tetras, described by Eigenmann et al. (1907). Of complex taxonomy, for a long time, some species belonging to the genus were considered *incertae sedis* in the family Characidae (Lima, 2003). Morphological features that are diagnostic indicators of the genus are also found in some species historically attributed to the genus *Astyanax*, and some studies over the years have shown that these species are phylogenetically closer to *Deuterodon* species than to other species of *Astyanax*, like *Astyanax giton*, *Astyanax hastatus*, *Astyanax intermedius, Astyanax ribeirae, Astyanax taeniatus* among others (Lucena and Lucena, 1992; Silva et al., 2017; Terán et al., 2020).

After an extensive revision, conducted by Terán et al. (2020), several coastal species of *Astyanax*, including the species mentioned in the paragraph above, as well as *Hyphessobrycon luetkenii*, were transferred to the genus *Deuterodon*. In addition, the authors also proposed that *Myxiopis* and *Probolodus* were synonyms of *Deuterodon*. Currently, the genus includes 24 valid species, whereas *Deuterodon potaroensis* remains as *incertae sedis* (Fricke et al., 2022). Of these, nine have described karyotypes (Table 1) and 20 have sequences deposited in GenBank, four of which are of the *ATP synthase subunit 6* gene (Benson et al., 2015). Among *Deuterodon*, *Deuterodon hastatus* constitutes a species complex (Kavalco et al., 2009), which was identified based on karyotype differences between populations, biological limits between specimens, and the absence of hybridism (Kavalco *et al.*, 2009; Pazza et al., 2018). 

**Table 1 -:** Cytogenetic characteristics of nine *Deuterodon* species.

Species	Sample procedence	2n	Karyotypic formulae	FN	C-banding	Ag-NORs	GC-rich sites	18S	5S	As-51	References
*D. giton* ^*a*^	Jacuí stream/SP - Paraíba do Sul river basin	50	6M+8SM+8ST+28A	72	Pericentromeric and interstitial. Many chromosomes.	6	absent	10	10	absent	Kavalco and Moreira-Filho (2003) Kavalco et al. (2004) Kavalco *et al*. (2007)
	Latão Creek/MG Doce river basin	50	6M+8SM+24ST+12A	88	Multiple markes many chromosomes.	2	2	10	2	-	Aguiar et al. (2011)
*D. intermedius* ^*a*^	Jacuí stream/SP - Paraíba do Sul river basin	50	6M+8SM+4ST+32A	68	Pericentromeric and interstitial. Many chromosomes.	6	absent	12	10	absent	Kavalco and Moreira-Filho (2003) Kavalco et al. (2004) Kavalco *et al*. (2007)
*D. hastatus* ^*a*^	Ypiranga Community/RJ Guapimirim-Macacu river basin	50	4M+8SM+10ST+28A (Cytotype A)	72	Pericentromeric and interstitial. Many chromosomes.	3	-	6	-	absent	Kavalco et al. (2009)
	Santana de Japuíba County/RJ Guapimirim-Macacu river basin	50	8M+10SM+14ST+18A (Cytotype B)	82	Pericentromeric and interstitial. Many chromosomes.	3	-	8	-	absent	Kavalco et al. (2009)
	Macacu River Guapimirim-Macacu river basin	50	6M+8SM+4ST+32A (Cytotype C)	68	Pericentromeric and interstitial. Many chromosomes.	2	-	6	-	absent	Kavalco et al. (2009)
	Town of Chachoeiras de Macacu/RJ Guapimirim-Macacu river basin	50	6M+8SM+4ST+32A (Cytotype C)	68	Pericentromeric and interstitial. Many chromosomes.	2	-	6	-	absent	Kavalco et al. (2009)
	Angra dos Reis/RJ Ariró river basin	50	6M+8SM+8ST+28A (Cytotype D)	72	-	-	-	6	7	-	present work
*D. ribeirae* ^*a*^	Poço Grande Community, Iporanga City/SP Ribeira de Iguape river basin	50	4M+10SM+6ST+30A	70	Pericentromeric. Some chromosomes.	2	-	4	6	absent	Kavalco et al. (2010)
	Town of Registro/SP Ribeira de Iguape river basin	50	4M+10SM+6ST+30A	70	Pericentromeric. Some chromosomes.	2	-	4	6	absent	Kavalco et al. (2010)
	Town of Sete Barras/SP Ribeira de Iguape river basin	50	4M+10SM+6ST+30A	70	Pericentromeric. Some chromosomes.	-	6	4	6	absent	Kavalco et al. (2010)
*D. taeniatus* ^*a*^	Hydroelectric Risoleta Neves Reservoir/MG Doce river basin	50	14M+12SM+16ST+8A	92	Centromeric and pericentromeric. Most SM and ST.	4-8	-	10	8	-	Da Cunha et al. (2016)
		50	10M+14SM+18ST+8A	92	Centromeric and pericentromeric. Most SM and ST.	4-8	-	8	8	-	Da Cunha et al. (2016)
*D. pedri*	Sant’Anna dos Ferros/MG Santo Antônio river basin	50	12M+12SM+20ST+6A	94	Centromeric many chromosomes. Instersticial some chromosomes.	2-4	-	10	2	-	Coutinho-Sanches and Dergam (2015)
*D. iguape* ^*b*^	Ipiranga River/SP Ribeira do Iguape river basin	50	14M/SM+36ST/A	-	-	-	-	-	-	-	Portela *et al.* (1988)
*D. stigmaturus*	Maquiné River/RS Tramandaí river basin	50	8M+6SM+2ST+34A	66	Pericentromeric. All chromossomes.	4-7	Many A short arms.	8	-	-	Mendes *et al.* (2011)
*D. janeiroensis* ^*a*^	Açungui River/PR Ribeira do Igapé river basin	50	6M+14SM+14SM+16A	84	Centromeric and telomeric. Most chromosomes	3-7	16	22	2	14	Carvalho et al. (2002) Vicari et al. (2008)
	Sacovão River/PR Ribeira do Igapé river basin	50	6M+14SM+14SM+16A	84	Centromeric and telomeric. Most chromosomes	3-7	16	22	2	14	Vicari et al. (2008)

^a^ In the reference, named as *Astyanax*. ^b^ In the reference, it is named as *Deuterodon pedri*.

Kavalco et al. (2009) described three distinct cytotypes (A, B and C) of *D. hastatus* in the Guapimirim-Macacu river basin (Table 1). In addition to karyotype formulas, different combinations in the patterns of active nucleolar organizer regions (three or two sites) and *18S rDNA* sites (six or eight sites) can differ the cytotypes (Table 1), despite the number of chromosomes (2n = 50) and patterns of constitutive heterochromatin (few positive markings in pericentromeric regions) remaining conserved. Variations in the karyotype formula and different distribution patterns with *18S rDNA* were also reported for two sympatric karyomorphs of the species *Deuterodon taeniatus* (Cunha et al., 2016). In turn, variations in the karyotype formula can be observed among allopatric populations of *Deuterodon giton* from the Paraíba do Sul River (Kavalco and Moreira-Filho, 2003; Kavalco *et al.*, 2007) and Doce River basins, and for the latter, hybridization with individuals of *Oligosarcus argenteus* (Aguiar, 2011) was reported.

In the present study, we investigated the phylogenetic relationships between different populations of the *D. hastatus* species complex and other species distributed on the southeastern Brazilian coast, through an integrated approach comparing cytogenetic and molecular data. Moreover, we present a new cytotype of *D. hastatus*, named cytotype D. We also estimated the divergence time within the group, using a molecular clock analysis, and discuss the data considering both the karyotypic and molecular evolution of the group.

## Material and Methods

We analyzed cell suspensions from eight specimens of *D*. *hastatus* that were deposited in the Tissues and Suspension bank of the Federal University of Viçosa Campus Rio Paranaíba. The specimens were collected by Kavalco, K.F. in 2008, in the basin of the Ariró River (East Atlantic watershed) near the municipality of Angra dos Reis/RJ (W 22º54ʹ36.1”/S 44º19’50.3”), and were deposited in the ichthyological collection of the Museum of Zoology of the Federal University of Rio Grande do Sul (Universidade Federal do Rio Grande do Sul - UFRGS) after identification (at the time, designated as *Astyanax hastatus*), under the code USP 3665-3694.

The metaphase chromosomes were obtained using the protocol of Gold et al. (1990). We then characterized, from a morphological point of view, each chromosome type, according to the arm ratio proposed by Levan (1964). Subsequently, the *18S rDNA* (Hatanaka and Galetti, 2004) and *5S rDNA* (Martins and Galetti, 1999) probes were mapped to *D. hastatus* chromosomes via fluorescence in situ hybridization (Pinkel et al., 1986; modified by Pazza et al., 2006). The probes were labeled with biotin-14-dATP via nick translation using the BioNick labeling kit according to the manufacturer’s instructions (Invitrogen LT, Carlsbad, CA, USA).

We randomly chose six individuals from the sample population of *D. hastatus* for *ATP synthase subunit 6* sequencing. DNA from those individuals were isolated from liver tissue using the Purelink Genomic Kit extraction kit (Invitrogen LT), according to the manufacturer’s instructions. After DNA quantification, we diluted the aliquots to a working concentration of 10 ng/μL and amplified the *ATPase 6* sequences using the primers ATP 8.2_L8331 (5′-AAAGCRTYRGCCTTTTAAGC) and CO3.2_H9236 (5′-GTTAGTGGTCAKGGGCTTGGRTC) (Sivasundar et al., 2001).

**Figure 1 -:**
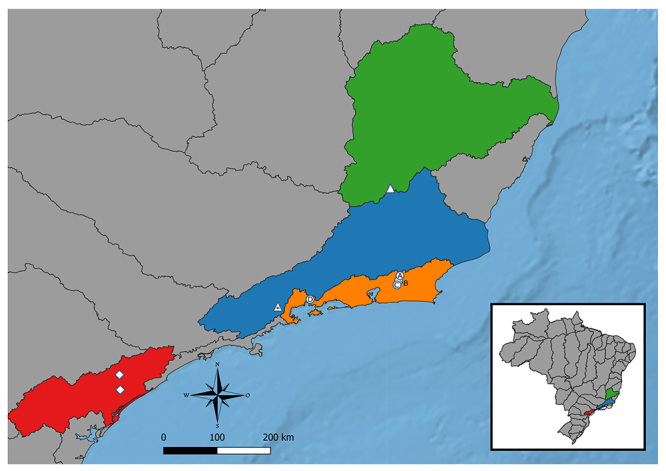
Map demonstrating the Deuterodon populations used in the phylogeny. Diamonds indicate populations of *D. ribeirae*, triangles indicate populations of *D. giton* (asterisk symbolizing sympatry with *D. intermedius*) and circles indicate populations of *D. hastatus* (letters representing the different cytotypes). In red the Ribeira de Iguape river basin, in blue the Paraíba do Sul river basin, in green the Doce river basin and in orange the coastal drainages of the state of Rio de Janeiro.

We used the MEGA-X software (Kumar et al., 2018) to perform sequence visualization, editing, and alignment, employing the MUSCLE algorithm (Edgar, 2004), in addition to calculating the interpopulation *p* distance (Thompson et al., 1994). For phylogenetic analyses, we employed the maximum likelihood estimation method, using IQ-TREE v2.1.2 software with 1000 ultrafast bootstrap replications (Minh et al., 2020). In our dataset, we included sequences of the *ATPase 6* gene from four specimens of *D. giton* (collected from basin streams of the Doce River and the Paraíba do Sul River), three specimens of *Deuterodon intermedius* (basin of Paraíba do Sul River), seven specimens of *Deuterodon ribeirae* (two localities in the basin of Ribeira de Iguape River), and 18 specimens of *D. hastatus* (four localities in the basin of the Guapimirim-Macacu River), in addition to the other 70 sequences from 24 species of the genera *Astyanax*, *Psalidodon*, *Roeboides*, *Bryconamericus*, *Eretmobrycon*, and *Triportheus* (outgroup) present in the NCBI database. The information regarding all sequences used is summarized in supplementary material Table S1. The geographic location of all *Deuterodon* populations used in our phylogeny can be seen in Figure 1. 

The divergence times of the group were estimated based on a Bayesian relaxed clock model, using BEAUti v. 2.6.6 and BEAST v. 2.6.6 software (Drummond and Bouckaert, 2015). The relaxed clock model used presented a log-normal distribution (non-correlated). For the “Tree Prior” parameter, we used the macroevolutionary Birth-Death model, and as a nucleotide substitution model, we used HKY+G, estimated using ModelFinder (Kalyaanamoorthy et al., 2017). We used four calibration points, which are as follows: 1) the fossil characid †*Paleotetra* spp. from the Eocene-Miocene (Weiss et al., 2012), used to limit the minimum age of the clade of all characids included in our analysis by implementing a log-normal prior offset of 33.9 million years ago, with a standard deviation of 1; 2) the fossil Triportheidae †*Lignobrycon ligniticus* from the late Oligocene (Woodward, 1898), used to limit the clade containing all *Triportheus* species included in our analysis by implementing a log-normal prior offset of 27.5 million years ago, with a standard deviation of 1; 3) the fossil characid †*Megacheirodon unicus* from the late Oligocene (Travassos and Santos, 1955), used to limit the clade containing all Stervadiinae (*Eretmobrycon* spp. + *Bryconamericus* spp.) in our analysis by implementing a lognormal prior offset of 27.5 million years ago, with a standard deviation of 1; 4) and finally, we used, as a calibration point, the origin of *Astyanax* species in Central America, dated by Ornelas-García et al. (2008) to 7.8-8.1 million years ago. This last calibration point was used to restrain the minimum age of the clade containing all *Astyanax* and *Psalidodon* species in our analysis by implementing a log-normal prior offset of 8 million years ago, with a standard deviation of 0.7. We constructed a haplotype network with the *Deuterodon* species, using the Haplotype Viewer software (Salzburger et al., 2011).

## Results

### Cytogenetics

**Figure 2 -:**
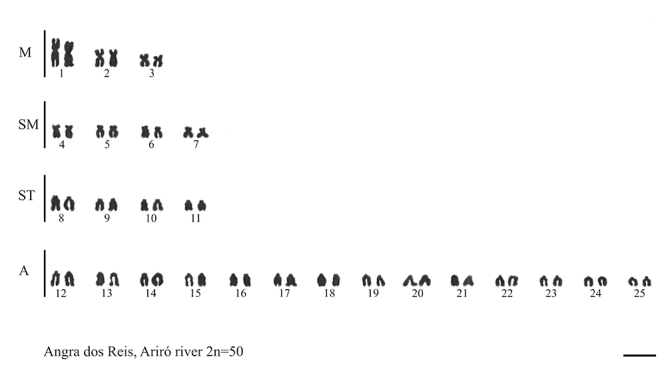
Karyotype of D. hastatus from the Ariró river, Angra dos Reis-RJ. Scale bar: 5 µm.

**Figure 3 -:**
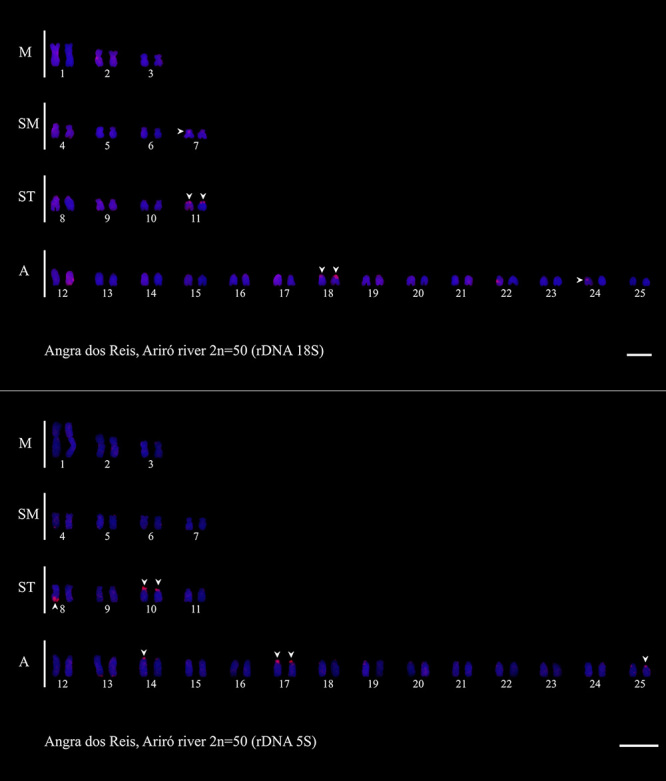
Karyotype of D. hastatus after FISH with 18S rDNA (A) and 5S rDNA (B) probes. Scale bar: 5 μm.

The population analyzed in this study presented 2n = 50 chromosomes, with the karyotype formula 6M+8SM+8ST+28A and NF = 72 (Figure 2). In relation to the *18S rDNA*, we observed subtelomeric markers on the short arms of chromosome pairs 11 and 18 and interstitial markers on one of the chromosomes of pairs 7 and 24. We also observed *5S rDNA* sites in the subtelomeric region of the short arms of chromosome pairs 10 and 17 and in the terminal region of one of the chromosomes of pairs 8, 14, and 25 (Figure 3).

### Phylogeny

**Figure 4 -:**
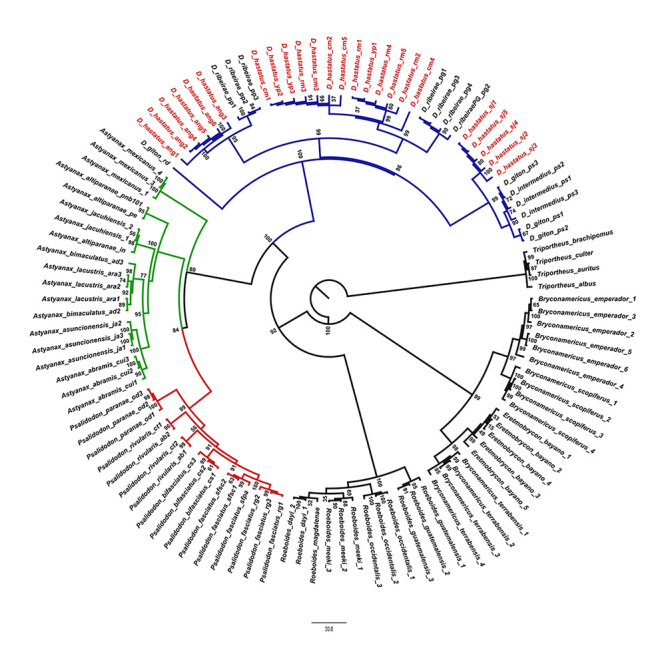
Phylogenetic tree of five populations of *D. hastatus*, two of *D. ribeirae*, two of *D. giton* and one of *D. intermedius* plus 70 sequences from 24 species outside the genus *Deuterodon*. Bootstrap values are demonstrated on internal nodes. The best evolutionary model, used in the analysis, was the TIM3+F+I+G4 according to ModelFinder.

**Figure 5 -:**
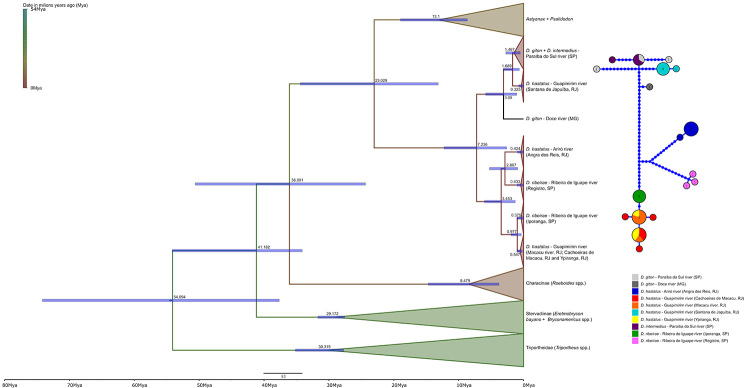
On the left, calibrated Bayesian tree, based on the *ATP synthase subunit 6* gene, demonstrating the evolutionary relationships between the species of *Deuterodon* analyzed plus outgroups. Timescale indicated on the x-axis in millions of years. Numbers on inner nodes represent divergence estimates and blue bars represent 95% highest posterior density interval. On the right, haplotype network of the four *Deuterodon* species analyzed, length of branches proportional to the number of mutations, number of sequences per haplotype demonstrated within each haplotype.

The phylogenetic analysis based on sequences of the *ATPase 6* region showed three clusters containing different populations of *D. hastatus* as follows: (1) one in which individuals from *D. hastatus* (cytotype D, presented in this study) from the Ariró River population, Angra dos Reis/RJ, were grouped with *D. ribeirae* (PP) from the Pesqueiro Paraíso, city of Registro/SP (bootstrap = 95 %); (2) one in which individuals of three populations of *D. hastatus*, two collected from the main channel of the Macacu river, in the municipality of Cachoeiras de Macacu (cytotype C *sensu* Kavalco et al., 2009) and one in the community Ypiranga (cytotype A *sensu* Kavalco *et al.*, 2009), from the basin of the Guapimirim-Macacu/RJ River, are grouped with individuals of *D. ribeirae* (PG) from the community of Poço Grande, Iporanga/SP (bootstrap = 99%); and finally, (3) on in which the sequences from individuals of *D. hastatus* from Santana de Japuíba population (cytotype B *sensu* Kavalco *et al.*, 2009) were found to be more closely related to those of *D. intermedius* and *D. giton* from the region of Cunha/SP, basin of the Paraíba do Sul River (bootstrap = 99%). In the maximum likelihood phylogram, *D. giton* from the basin of the Doce River diverged before the clade that harbored the other *Deuterodon* species used in the analysis (Figure 4). Nevertheless, the same phenomenon was not observed in the Bayesian tree from the molecular clock analysis (Figure 5).

According to the molecular clock analysis, the *Deuterodon* group diverged from the clade containing *Astyanax* and *Psalidodon* at some point in the Oligocene-to-Miocene transition, approximately 23 million years ago (95% HPD, 13.1-34.4 Mya). The first divergence among the analyzed *Deuterodon* species occurred approximately 7.2 million years ago (95% HPD, 2.6-12.6 Mya), between the Miocene and Pliocene, and it was during this period that the lineage of the population identified as *D. hastatus* in Santana de Japuíba (cytotype B), together with *D. giton* and *D. intermedius*, diverged from the other populations of *D. hastatus* and *D. ribeirae*. The separation of *D. hastatus* of Santana de Japuíba from *D. giton* and *D. intermedius* found in the Paraíba do Sul River occurred approximately 1.7 million years ago (95% HPD, 0.6-3.1 Mya), during the Pliocene-to-Pleistocene transition. In relation to the other populations of *D. hastatus* and *D. ribeirae*, the first divergence occurred between the Pliocene and Pleistocene, approximately 3.4 million years ago (1.3-6 Mya), and was responsible for separating the populations of *D. hastatus* from the basin of the Guapimirim-Macacu River (cytotypes A and C) and *D. ribeirae* of Iporanga (SP) from *D. hastatus* of the Ariró River (cytotype D) and *D. ribeirae* of Registro (SP). The final branching between the remaining lineages of *D. hastatus* of the Guapimirim-Macacu River and *D. ribeirae* found in Iporanga occurred approximately 1 million years ago (95% HPD, 0.3-1.9 Mya), during the Pleistocene, and that between *D. hastatus* found in Ariró River and *D. ribeirae* collected in Registro occurred approximately 2.9 million years ago (95% HPD, 0.9-5.3 Mya) between the Pliocene and Pleistocene.

We obtained 18 haplotypes from the four species of *Deuterodon*, the polymorphic sites (S) were 83, the nucleotide diversity (Pi) was 0.04476 and the haplotype diversity (Hd) was 0.936. In the haplotype network (Figure 5), it was possible to observe at least four major haplogroups among the *Deuterodon* lineages. However, these haplogroups did not correspond at all to the taxonomic names of the four studied species. The first haplogroup (dark green, orange, red and yellow) was found to include three populations of *D. hastatus* (cytotypes A and C) found in the Guapimirim-Macacu River (Macacu River, RJ; Cachoeiras de Macacu, RJ; and Ypiranga community, RJ) and *D. ribeirae* collected from the Ribeira de Iguape River (Iporanga, SP). Seven haplotypes were found to form this haplogroup, with all being very close to each other. The second haplogroup (pink) was composed of only three haplotypes of *D. ribeirae* from the Ribeira de Iguape River (Registro, SP). The third haplogroup (dark blue) was composed of two haplotypes of *D. hastatus* from the Ariró River (cytotype D, Angra dos Reis, RJ). The fourth haplogroup (light gray, dark gray, light blue and purple) was composed of four haplotypes of *D. intermedius* and *D. giton* from the Paraíba do Sul River (Cunha, SP) and two haplotypes of *D. hastatus* from the Guapimirim-Macacu River (cytotype B, Santana de Japuiba, RJ). One of these haplotypes is shared by *D. intermedius* and *D. giton* from the rio Paraíba do Sul, with two other haplotypes from each of these species being derived from this one. Another haplotype of *D. giton* from the Paraíba do Sul river and two other haplotypes of *D. hastatus* from Santana de Japuíba seem to diverge from this first one from an ancestral haplotype. The genetic distances among all analyzed *Deuterodon* populations are summarized in Table 2.

**Table 2 -:** Bottom triangle: genetic distances between analytical populations. Upper triangle: standard deviations.

	1.	2.	3.	4.	5.	6.	7.	8.	9.	10.
1. *D. giton* - Paraíba do Sul river (Cunha, SP)		0.00584	0.01007	0.01137	0.01135	0.00588	0.01133	0.00364	0.01089	0.01214
2. *D. giton* - Doce river (Latão Creek, MG)	0.02511		0.01071	0.01091	0.01088	0.00611	0.01084	0.00514	0.01035	0.01195
3. *D. hastatus* - Ariró river (Angra dos Reis, RJ)	0.08014	0.06560		0.00766	0.00750	0.01135	0.00756	0.01075	0.00750	0.00916
4. *D. hastatus* - Guapimirim river (Cachoeiras de Macacu, RJ)	0.07721	0.06215	0.03773		0.00125	0.01259	0.00111	0.01187	0.00290	0.00786
5. *D. hastatus* - Guapimirim river (rio Macacu, RJ)	0.07684	0.06177	0.03584	0.00218		0.01251	0.00111	0.01185	0.00257	0.00802
6. *D. hastatus* - Guapimirim river (Santana de Japuiba, RJ)	0.02109	0.02298	0.07916	0.07382	0.07345		0.01246	0.00503	0.01196	0.01241
7. *D. hastatus* - Guapimirim river (Ypiranga, RJ)	0.07596	0.06089	0.03672	0.00201	0.00113	0.07257		0.01176	0.00273	0.00780
8. *D. intermedius* - Paraíba do Sul river (Cunha, SP)	0.01381	0.01820	0.07627	0.07282	0.07244	0.01281	0.07156		0.01137	0.01205
9. *D. ribeirae* - Ribeira de Iguape river (Iporanga, SP)	0.07345	0.05838	0.03547	0.00603	0.00414	0.07006	0.00502	0.06905		0.00799
10. *D. ribeirae* - Ribeira de Iguape river (Registro, SP)	0.08475	0.06968	0.04488	0.04746	0.04557	0.07947	0.04645	0.08035	0.04143	

## Discussion

In this work, we describe a new cytotype of *D. hastatus* based on a study of a population from Ariró River, Angra dos Reis/RJ. We referred to it as cytotype D, showing the extensive range of karyotype variation in this group. Herein, we also present the first data of the physical mapping of *5S rDNA* in *D. hastatus*. Our phylogeny also showed paraphyletic groups in at least three species of *Deuterodon*, namely *D. hastatus*, *D. ribeirae*, and *D. giton*, suggesting the existence of cryptic species in these groups. We also provide the first proposal of a molecular clock for the genus and demonstrate that the evolution of *Deuterodon* in southeastern Brazil was strongly influenced by the neotectonic events that marked the Pliocene-to-Pleistocene transition.

Despite differences in the karyotype formula (Table 1), cytotype D of *D. hastatus* has the same number of *18S rDNA* sites (six) as the other cytotypes of the species, with the exception of cytotype B from Santana de Japuíba, which has eight sites (Kavalco et al., 2009). In addition, the genetic distance of this cytotype to the others is 3.67% for cytotype A, 7.9% for cytotype B and 3.58-3.77% for cytotype C (Table 2). If we integrate the observed phylogenetic, karyotypic and genetic distances data, we can conclude the existence of at least three different evolutionary significantly units (ESUs) in *D. hastatus*: one composed of specimens of cytotypes A and C, which have genetic distances of 0.1 and 0.2% between them, another composed by cytotype B, whose genetic distance in relation to the others is between 7.2% and 7.9%, and finally another composed by cytotype D presented here.

As reported for *D. giton*, *D. intermedius*, and *D. ribeirae* (Kavalco and Moreira-Filho, 2003; Kavalco *et al.*, 2007, 2010), *D. hastatus* lacks the marker chromosome, a metacentric pair carrying the *5S rDNA* site in the pericentromeric region, identified by Kavalco *et al.* (2004, 2010) as a common feature of the other Stethaprioninae genera present in the continent, and first described for some *Astyanax* species by Almeida-Toledo et al. (2002). Our result corroborates the hypothesis of Pazza et al. (2018) that coastal Stethaprioninae, diverged before the emergence of this site in the interstitial position (i.e., before the fixation of the marker chromosome). The large interspecific variation in *5S rDNA* of the *Deuterodon* species which this marker is disponible is also remarkable. The studied population of *D. hastatus* possess three more *5S rDNA* sites than *D. ribeirae* (Kavalco *et al.*, 2010), three site less than *D. giton* found in the Paraíba do Sul River (Kavalco *et al.*, 2004), five more than *D. giton* from the Doce River (Aguiar, 2011), three less than *D. intermedius* (Kavalco *et al.*, 2004), and one fewer than *D. taeniatus* (Da Cunha et al., 2016). The first studies based on this ribosomal DNA sites seemed to indicate a conserved pattern in Characiformes. However, the idea that it is a homogeneous marker in fish was certainly due to the low representativeness in front of the high number of existing species. Even for *Deuterodon*, it is still necessary to analyze species mainly from the southern Atlantic and northeastern Brazilian coasts and from Guyana, since chromosomal data are concentrated on populations from the southeastern Brazilian coast (Table 1).

According to Pazza et al. (2018), species of the genus *Deuterodon* (referred to as *Astyanax* Clade 1) are known to present some symplesiomorphic cytogenetic features, such as the diploid number of 2n = 50, a low FN (FN = 66 - 84), and up to 10 *5S rDNA* sites, all located in the terminal region of the chromosomes. All of these features can be observed in different *Deuterodon* species, except for the low FN, since the populations of the genus in Minas Gerais state are likely to have a high FN (FN = 88-94, Table 1) (Aguiar, 2011; Coutinho-Sanches and Dergam, 2015; Da Cunha et al., 2016). Another feature that was mentioned is the absence of a positive hybridization signal for *As51* satellite DNA in many species of the genus (Kavalco et al., 2009), except for *Deuterodon janeiroensis* (Carvalho et al., 2002; Vicari et al., 2008a).

The cytotype B of *D. hastatus* from the city of Santana de Japuíba had already been suggested by Kavalco et al. (2009) to be a possible cryptic species of *D. hastatus*, owing to its karyotype differences. As observed by Pazza et al. (2018), this population was closer to *D. giton* and *D. intermedius* than to *D. ribeirae* and the other populations of *D. hastatus* (Figures 4 and 5). In addition to differences in the karyotype formula, this population was also found to have a higher number of *18S rDNA* sites than *D. hastatus* and *D. ribeirae* (8 sites vs 6 and 4 respectively) but a lower number than *D. giton* and *D. intermedius* (which have 10 and 12 respectively) (Table 1). Thus, our phylogram (Figure 4) can be separated into two subclades, one characterized by *Deuterodon* populations with a stable number of six *18S rDNA* sites (*D. hastatus* and *D. ribeirae*) and the other with a variable number of eight or more *18S rDNA* sites (*D. hastatus* SJ, *D. giton*, and *D. intermedius*).

Another finding was the absence of monophyly from *D. giton* haplotypes, as *D. giton* from the Paraíba do Sul River is paraphyletic in relation to *D. intermedius* and *D. giton* from the Doce River does not belong to this group. These specimens were also used in cytogenetic analyses, and in addition to the discrepant karyotype formulas (Table 1), the population from Paraíba do Sul River had more number of active NORs (Kavalco and Moreira-Filho, 2003) and *5S rDNA* sites (Kavalco *et al.*, 2004) than the population of the Doce River (Aguiar, 2011) (Table 1). Therefore, we propose that the population of the Doce River, owing to its molecular and cytogenetic features, is a different cryptic species from that found in the Paraíba do Sul River. Thus, it is very likely that the diversity observed in *D. hastatus*, composed of cryptic species, is similar to that found in *D. giton*.

The geographic distribution of the *D. hastatus* populations can be explained by vicariance events related to two hypotheses, which are non-mutually exclusive. One is the capture of headwaters of one river by another that could lead to the fixation of different karyomorphs in nearby locations (Vicari et al., 2008 b ). This hypothesis seems to fit well with the karyotype and molecular differentiation of *D. hastatus* found in the population of Santana de Japuíba in relation to other populations from the basin of the Guapimirim-Macacu River (Kavalco et al., 2009). The other hypothesis refers to sea level fluctuations that occurred in the Pleistocene, which might have led to the isolation of coastal basins. Radiation chronologically shaped by these fluctuations in the sea level has been proposed for the *Odontesthes perugiae* complex (Atheriniformes, Atherinopsidae) (Beheregaray et al., 2002). This second hypothesis seems to align more with the situation of *D. hastatus* from the Ariró River and the other populations of *D. hastatus* from the Guapimirim-Macacu River (Kavalco *et al.*, 2009), as well as with that of *D. giton* from the Paraíba do Sul and Doce Rivers (Kavalco and Moreira-Filho, 2003; Kavalco *et al*., 2007; Aguiar, 2011).

The oldest cladogenic events among *Deuterodon* species, observed in this study (7.2 Mya), seem to match the oldest cladogenic events among coastal fish of the genus *Mimagoniates*, estimated at 6.8 Mya (Camelier et al., 2018). These events coincide with strong tectonic activities that occurred in the Neogene, which caused fluvial capture by coastal drainages (Menezes et al., 2008; Ribeiro, 2006). The more recent cladogenic events, observed between *D. hastatus* from Santana de Japuíba and *D. giton*/*D. intermedius* from Paraíba do Sul River, between *D. hastatus* from Ariró River and *D. ribeirae* from Iporanga, and finally, between *D. hastatus* from Macacu/Ypiranga and *D. ribeirae* from Registro, seem to all date back to the transition epoch between the Pliocene and Pleistocene (1.7, 2.9, and 1 Mya, respectively). This date seems to coincide with the estimated divergence of *Astyanax lacustris* and *Astyanax altiparanae* species (Cunha et al., 2019) and with coastal *Oligosarcus* species in southeastern and southern Brazil (Wendt et al., 2019). This epoch was characterized by intense tectonic activities, which caused several drainage rearrangements through stream capture events and marine transgressions (Ribeiro, 2006).

The successive marine regressions that occurred in the Pliocene-to-Pleistocene transition were associated with frequent fauna exchanges between Brazilian coastal basins (Wendt et al., 2019). Thus, the most recent cladogenic events among *Deuterodon* from southeastern Brazil can be explained by several exchanges of fauna between the river basins of Ribeira de Iguape, Paraíba do Sul, Doce and coastal rivers of the State of Rio de Janeiro, such as the basin of the Guapimirim-Macacu River (Figure 1). Thus, it is possible that the phylogeographic patterns observed in this study could be explained by events comprising the reciprocal migration of *D. ribeirae* to the Guapimirim-Macacu basin and *D. hastatus* to the basin of Ribeira de Iguape. This would explain not only the apparent taxonomic confusion between the two species but also the groups of populations between these two basins, rather than populations within each basin. A similar pattern can be seen in the phylogeny of *Mimagoniates macrolepis*, where a sample from Itanhaém, SP, is closer to one from the Macacu River, RJ, than to another sample from the Ribeira de Iguape basin, SP (Camelier et al., 2018).

In this work, we were able to observe, based on both cytogenetic and molecular data, the genetic and chromosomal diversity present in some *Deuterodon* species, even at the intraspecific level. We corroborated our hypothesis referring to the existence of cryptic species complexes in the genus, such as *D. hastatus* and *D. giton*. Further studies involving taxonomic and morphological analyses are required to formally classify and describe these units as new species.

## Supplementary material

Table S1 - Data access.

## References

[B1] Aguiar HJACD (2011). First report on spontaneous hybridization between *Astyanax giton* Baird & Girard 1854 and *Oligosarcus argenteus* Günther 1864 (Pisces: Characidae): Ecological and phylogenetic inferences.

[B2] Almeida-Toledo LF, Ozouf-Costaz C, Foresti F, Bonillo C, Porto-Foresti F, Daniel-Silva MFZ (2002). Conservation of the 5S-bearing chromosome pair and co-localization with major rDNA clusters in five species of Astyanax (Pisces, Characidae). Cytogenet Genome Res.

[B3] Beheregaray LB, Sunnucks P, Briscoe DA (2002). A rapid fish radiation associated with the last sea-level changes in southern Brazil: The silverside Odontesthes perugiae complex. Proc Biol Sci.

[B4] Benson DA, Clark K, Karsch-Mizrachi I, Lipman DJ, Ostell J, Sayers EW (2015). GenBank. Nucleic Acids Res.

[B5] Camelier P, Menezes NA, Costa-Silva GJ, Oliveira C (2018). Molecular phylogeny and biogeographic history of the Neotropical tribe Glandulocaudini (Characiformes: Characidae: Stevardiinae). Neotrop Ichthyol.

[B6] Carvalho ML, Oliveira C, Foresti F (2002). Cytogenetic analysis of five species of the subfamily Tetragonopterinae (Teleostei, Characiformes, Characidae). Caryologia.

[B7] Coutinho-Sanches N, Dergam JA (2015). Cytogenetic and molecular data suggest Deuterodon pedri Eigenmann, 1907 (Teleostei: Characidae) is a member of an ancient coastal group. Zebrafish.

[B8] Cunha MS, Reis VJC, Dergam JA (2016). Closely related syntopic cytotypes of Astyanax taeniatus (Jenyns, 1842) from the upper Piranga River, upper Doce Basin in southeastern Brazil. Zebrafish.

[B9] Cunha MS, Fregonezi AR, Fava L, Hilsdorf AW, Campos LA, Dergam JA (2019). Phylogeography and historical biogeography of the Astyanax bimaculatus species complex (Teleostei: Characidae) in coastal southeastern South America. Zebrafish.

[B10] Drummond AJ, Bouckaert RR (2015). Bayesian Evolutionary Analysis with BEAST.

[B11] Edgar RC (2004). MUSCLE: Multiple sequence alignment with high accuracy and high throughput. Nucleic Acids Res.

[B12] Eigenmann CH, McAtee WL, Ward PD (1907). On further collections of fishes from Paraguay. American Carnegie Mus.

[B13] Fricke R, Eschmeyer WN, Van der Laan R (2022). Eschmeyer’s Catalog of Fishes: Genera, species, references. California Academy of Sciences.

[B14] Gold JR, Li YC, Shipley NS, Powers PK (1990). Improved methods for working with fish chromosomes with a review of metaphase chromosome banding. J Fish Biol.

[B15] Hatanaka T, Galetti PM (2004). Mapping of the 18S and 5S ribosomal RNA genes in the fish Prochilodus argenteus Agassiz, 1829 (Characiformes, Prochilodontidae). Genetica.

[B16] Kalyaanamoorthy S, Minh BQ, Wong TK, Von Haeseler A, Jermiin LS (2017). ModelFinder: Fast model selection for accurate phylogenetic estimates. Nat Methods.

[B17] Kavalco KF, Moreira- O (2003). Cytogenetical analyses in four species of the genus Astyanax (Pisces, Characidae) from Paraíba do Sul river basin. Caryologia.

[B18] Kavalco KF, Pazza R, Bertollo LAC, Moreira- O (2004). Gene mapping of 5S rDNA sites in eight fish species from the Paraíba do Sul river basin, Brazil. Cytogenet Genome Res.

[B19] Kavalco KF, Pazza R, Bertollo LAC, Moreira- O (2007). Satellite DNA sites in four species of the genus Astyanax (Teleostei, Characiformes). Genet Mol Biol.

[B20] Kavalco KF, KdO Brandão, Pazza R, Almeida-Toledo LF (2009). Astyanax hastatus Myers, 1928 (Teleostei, Characidae): A new species complex within the genus Astyanax?. Genet Mol Biol.

[B21] Kavalco KF, Pazza R, de Almeida-Toledo LF (2010). Molecular cytogenetics of Astyanax ribeirae (Teleostei, Characidae), an endemic characin of the Atlantic rainforest. Nucleus (India).

[B22] Kumar S, Stecher G, Li M, Knyaz C, Tamura K (2018). MEGA X: Molecular evolutionary genetics analysis across computing platforms. Mol Biol Evol.

[B23] Levan A (1964). Nomenclature for centromeric position on chromosomes. Hereditas.

[B24] Lima FCT, Reis RE, Kullander SO, Ferraris CJ (2003). Check List of the Freshwater Fishes of South and Central America.

[B25] Lucena ZD, Lucena CD (1992). Revisão das espécies do gênero Deuterodon Eigenmann, 1907 dos sistemas costeiros do sul do Brasil com a descrição de quatro espécies novas (Ostariophysi, Characiformes, Characidae). Comun Mus Ciênc Tecnol PUCRS, Sér Zool.

[B26] Martins C, Galetti PM (1999). Chromosomal localization of 5S rDNA genes in Leporinus fish (Anostomidae, Characiformes). Chromosome Res.

[B27] Menezes NA, Ribeiro AC, Weitzman S, Torres RA (2008). Biogeography of Glandulocaudinae (Teleostei: Characiformes: Characidae) revisited: Phylogenetic patterns, historical geology and genetic connectivity. Zootaxa.

[B28] Minh BQ, Schmidt HA, Chernomor O, Schrempf D, Woodhams MD, Von Haeseler A, Lanfear R (2020). IQ-TREE 2: New models and efficient methods for phylogenetic inference in the genomic era. Mol Biol Evol.

[B29] Ornelas-García CP, Domínguez-Domínguez O, Doadrio I (2008). Evolutionary history of the fish genus Astyanax baird & Girard (1854) (Actinopterygii, Characidae) in Mesoamerica reveals multiple morphological homoplasies. BMC Evol Biol.

[B30] Pazza R, Kavalco KF, Bertollo LAC (2006). Chromosome polymorphism in Astyanax fasciatus (Teleostei, Characidae). 1. Karyotype analysis, Ag-NORs and mapping of the 18S and 5S ribosomal genes in sympatric karyotypes and their possible hybrid forms. Cytogenet Genome Res.

[B31] Pazza R, Dergam JA, Kavalco KF (2018). Trends in karyotype evolution in Astyanax (Teleostei, Characiformes, Characidae): Insights from molecular data. Front Genet.

[B32] Ribeiro AC (2006). Tectonic history and the biogeography of the freshwater fishes from the coastal drainages of eastern Brazil: An example of faunal evolution associated with a divergent continental margin. Neotrop Ichthyol.

[B33] Pinkel D, Straume T, Gray JW (1986). Cytogenetic analysis using quantitative, high-sensitivity, fluorescence hybridization. Proc Natl Acad Sci U S A.

[B34] Salzburger W, Ewing GB, von Haeseler A (2011). The performance of phylogenetic algorithms in estimating haplotype genealogies with migration. Mol Ecol.

[B35] Silva PC, Malabarba MC, Malabarba LR (2017). Using ancient DNA to unravel taxonomic puzzles: The identity of Deuterodon pedri (Ostariophysi: Characidae). Neotrop Ichthyol.

[B36] Sivasundar A, Bermingham E, Ortí G (2001). Population structure and biogeography of migratory freshwater fishes (Prochilodus: Characiformes) in major South American rivers. Mol Ecol.

[B37] Terán GE, Benitez MF, Mirande JM (2020). Opening the Trojan horse: Phylogeny of Astyanax, two new genera and resurrection of Psalidodon (Teleostei: Characidae). Zool J Linnean Soc.

[B38] Thompson JD, HIiggins DG, Gibson TJ (1994). CLUSTALW: Improving the sensitivity of progressive multiple sequence through weighing, position-speciﬁc gap penalties and weight matrix choice. Nucleic Acids Res.

[B39] Travassos H, Santos RS (1955). Caracídeos fósseis da Bacia do Paraíba. An Acad Brasil Ciênc.

[B40] Vicari MR, Artoni RF, Moreira O, Bertollo LAC (2008). Colocalization of repetitive DNAs and silencing of major rRNA genes. A case report of the fish Astyanax janeiroensis. Cytogenet Genome Res.

[B41] Vicari MR, Noleto RB, Artoni RF, Moreira- O, Bertollo LAC (2008). Comparative cytogenetics among species of the Astyanax scabripinnis complex: Evolutionary and biogeographical inferences. Genet Mol Biol.

[B42] Weiss FE, Malabarba LR, Malabarba MC (2012). Phylogenetic relationships of Paleotetra, a new characiform fish (Ostariophysi) with two new species from the Eocene-Oligocene of south-eastern Brazil. J Syst Palaeotol.

[B43] Wendt EW, Silva PC, Malabarba LR, Carvalho TP (2019). Phylogenetic relationships and historical biogeography of Oligosarcus (Teleostei: Characidae): Examining riverine landscape evolution in southeastern South America. Mol Phylogenet Evol.

[B44] Woodward AS (1898). Considerações sobre alguns peixes Terciários dos schistos de Taubaté, Estado de São Paulo, Brasil. Rev Mus Paulista.

