# Supplementary material for: Phylogeography and karyotypic evolution of some Deuterodon species from southeastern Brazil (Characiformes, Characidae, Stethaprioninae)
Source: Genet Mol Biol. 2023 Jul 10;46(3):e20230044. doi: 10.1590/1678-4685-GMB-2023-0044 (PMC10337518; doi:10.1590/1678-4685-GMB-2023-0044)
Supplement: Table S1 - [file 1415-4757-GMB-46-3-e20230044-s1.pdf]

Genetics and Molecular Biology
https://doi.org/10.1590/1678-4685-GMB-2023-0044

Supplementary Material to "Phylogeography and karyotypic evolution of some *Deuterodon* species from southeastern Brazil (Characiformes, Characidae, Stethaprioninae)"

Table S1 - Data access.

Species	Locality	ID	Accession number
Deuterodon hastatus	Ariró river / Angra dos Reis (RJ)	ang1	MH158929.1
D. hastatus	Ariró river / Angra dos Reis (RJ)	ang2	MH158930.1
D. hastatus	Ariró river / Angra dos Reis (RJ)	ang3	MH158931.1
D. hastatus	Ariró river / Angra dos Reis (RJ)	ang4	MH158932.1
D. hastatus	Ariró river / Angra dos Reis (RJ)	ang5	MH158933.1
D. hastatus	Ariró river / Angra dos Reis (RJ)	ang6	MH158934.1
D. hastatus	Guapimirim river / Santana de Japuíba (RJ)	pf1	MH158935.1
D. hastatus	Guapimirim river / Santana de Japuíba (RJ)	pf2	MH158936.2
D. hastatus	Guapimirim river / Santana de Japuíba (RJ)	pf3	MH158937.3
D. hastatus	Guapimirim river / Santana de Japuíba (RJ)	pf4	MH158938.4
D. hastatus	Guapimirim river / Santana de Japuíba (RJ)	pf5	MH158939.5
D. hastatus	Guapimirim river / Cachoeiras de Macacu (RJ)	pr1	MH158940.1
D. hastatus	Guapimirim river / Cachoeiras de Macacu (RJ)	pr2	MH158941.2
D. hastatus	Guapimirim river / Cachoeiras de Macacu (RJ)	pr3	MH158942.3
D. hastatus	Guapimirim river / Cachoeiras de Macacu (RJ)	pr4	MH158943.4
D. hastatus	Guapimirim river / Cachoeiras de Macacu (RJ)	pr5	MH158944.5
D. hastatus	Guapimirim river / Macacu river (RJ)	rm1	MH158945.1
D. hastatus	Guapimirim river / Macacu river (RJ)	rm2	MH158946.1
D. hastatus	Guapimirim river / Macacu river (RJ)	rm3	MH158947.1
D. hastatus	Guapimirim river / Macacu river (RJ)	rm4	MH158948.1
D. hastatus	Guapimirim river / Macacu river (RJ)	rm5	MH158949.1
D. hastatus	Guapimirim river / Ypiranga community (RJ)	yp1	MH158950.1
D. hastatus	Guapimirim river / Ypiranga community (RJ)	yp2	MH158951.1
D. hastatus	Guapimirim river / Ypiranga community (RJ)	yp3	MH158952.1
Deuterodon ribeirae	Ribeira de Iguape river / Iporanga (SP)	pg1	MH158975.1
D. ribeirae	Ribeira de Iguape river / Iporanga (SP)	pg2	MH158976.1
D. ribeirae	Ribeira de Iguape river / Iporanga (SP)	pg3	MH158977.1
D. ribeirae	Ribeira de Iguape river / Iporanga (SP)	pg4	MH158978.1
D. ribeirae	Ribeira de Iguape river / Registro (SP)	pp1	MH158979.1
D. ribeirae	Ribeira de Iguape river / Registro (SP)	pp2	MH158980.1

Species	Locality	ID	Accession number
D. ribeirae	Ribeira de Iguape river / Registro (SP)	pp3	MH158981.1
Deuterodon giton	Paraiba do Sul river / Cunha (SP)	ps1	-
D. giton	Paraiba do Sul river / Cunha (SP)	ps2	MH158927.1
D. giton	Paraiba do Sul river / Cunha (SP)	ps3	MH158928.1
Deuterodon intermedius	Paraiba do Sul river / Cunha (SP)	ps1	MH158953.1
D. intermedius	Paraiba do Sul river / Cunha (SP)	ps2	MH158954.1
D. intermedius	Paraiba do Sul river / Cunha (SP)	ps3	-
D. giton	Doce river / Latão Creek (MG)	rd	MF805815.1
Astyanax mexicanus	-	1	MH158964.1
A. mexicanus	-	3	MH158966.1
A. mexicanus	-	4	MH158967.1
Astyanax altiparanae	-	in	MH158821.1
A. altiparanae	-	pe	MH158822.1
A. altiparanae	-	pnb101	MH158823.1
Astyanax jacuhiensis	-	1	MH158955.1
A. jacuhiensis	-	2	MH158956.1
Astyanax bimaculatus	-	ad2	MH158857.1
A. bimaculatus	-	ad3	MH158858.1
Astyanax abramis	-	cui1	MH158811.1
A. abramis	-	cui2	MH158812.1
A. abramis	-	cui3	MH158813.1
Astyanax assuncionensis	-	ja1	MH158838.1
A. assuncionensis	-	ja2	MH158839.1
A. assuncionensis	-	ja3	MH158840.1
Astyanax lacustris	-	ara1	MH158957.1
A. lacustris	-	ara2	MH158958.1
A. lacustris	-	ara3	MH158959.1
Psalidodon paranae	-	od1	MH158969.1
P. paranae	-	od2	MH158970.1
P. paranae	-	od3	MH158971.1
Psalidodon rivularis	-	ab1	MH158982.1
P. rivularis	-	ab2	MH158983.1
P. rivularis	-	ct1	MH158987.1
P. rivularis	-	ct2	MH158988.1
Psalidodon bifasciatus	-	cs1	MH158848.1
P. bifasciatus	-	cs2	MH158849.1
P. bifasciatus	-	cs3	MH15885.10
Psalidodon fasciatus	-	rg1	KY118923.1
P. fasciatus	-	rg2	KY118924.1
P. fasciatus	-	rg3	KY118925.1
P. fasciatus	-	sfpa	KY118938.1
P. fasciatus	-	sfsc1	KY118939.1
P. fasciatus	-	sfsc2	KY1189.140
Roeboides dayi	-	1	AF040526.1
R. dayi	-	2	AF04052.17
Roeboides magdalenae	-	-	AF040525.1

Species	Locality	ID	Accession number
Roeboides meeki	-	1	AF040522.1
R. meeki	-	2	AF040523.1
R. meeki	-	3	AF040524.1
Roeboides occidentalis	-	1	AF040498.1
R. occidentalis	-	2	AF040499.1
R. occidentalis	-	3	AF040450.1
Roeboides guatemalensis	-	1	AF040490.1
R. guatemalensis	-	2	AF040491.1
R. guatemalensis	-	3	AF040492.1
Eretmobrycon bayano	-	1	AF412573.1
E. bayano	-	2	AF412574.1
E. bayano	-	3	AF412575.1
E. bayano	-	4	AF412576.1
E. bayano	-	5	AF412577.1
Bryconamericus terrabensis	-	1	AF412587.1
B. terrabensis	-	2	AF412588.1
B. terrabensis	-	3	AF412589.1
B. terrabensis	-	4	AF412590.1
Bryconamericus scopiferus	-	1	AF412627.1
B. scopiferus	-	2	AF412626.1
B. scopiferus	-	3	AF412625.1
B. scopiferus	-	4	AF412624.1
Bryconamericus emperador	-	1	AF412591.1
B. emperador	-	2	AF412592.1
B. emperador	-	3	AF412593.1
B. emperador	-	4	AF412612.1
B. emperador	-	5	AF412613.1
B. emperador	-	6	AF412614.1
Triportheus albus	-	-	MF188225.1
Triportheus auritus	-	-	MF188228.1
Triportheus culter	-	-	MF188229.1
Triportheus brachipomus	-	-	MF188231.1
